# Propionate catabolism by CD-associated adherent-invasive *E. coli* counteracts its anti-inflammatory effect

**DOI:** 10.1080/19490976.2020.1839318

**Published:** 2021-03-26

**Authors:** Allison Agus, Damien Richard, Tiphanie Faïs, Emilie Vazeille, Mélissa Chervy, Virginie Bonnin, Guillaume Dalmasso, Jérémy Denizot, Elisabeth Billard, Richard Bonnet, Anthony Buisson, Nicolas Barnich, Julien Delmas

**Affiliations:** a Inserm U1071, USC-INRAE 2018, Microbes, Intestin, Inflammation Et Susceptibilité De l’Hôte (M2ISH), Centre De Recherche En Nutrition Humaine Auvergne University Clermont Auvergne, Clermont-Ferrand, France; b INRAE, AgroParisTech, Micalis Institute, University Paris-Saclay, Jouy-en-Josas, France; c Department of Pharmacology, University Hospital of Clermont-Ferrand, France; d Department of Bacteriology, University Hospital of Clermont-Ferrand, France; e Service d’Hépato-Gastro Entérologie, 3iHP, CHU Clermont-Ferrand, Clermont-Ferrand, France; f Institut Universitaire De Technologie, University Clermont Auvergne, Clermont-Ferrand, France

**Keywords:** Crohn’s disease, adherent-invasive *E. coli*, propionate, methyl-citrate pathway, GPR43 agonist treatment

## Abstract

Crohn’s disease (CD) is a chronic and disabling inflammatory disorder of the gut that is profoundly influenced by intestinal microbiota composition, host genetics and environmental factors. Several groups worldwide have described an imbalance of the gut microbiome composition, called dysbiosis, in CD patients, with an increase in *Proteobacteria* and *Bacteroidetes* and a decrease in *Firmicutes*. A high prevalence of adherent-invasive *Escherichia coli* (AIEC) pathobionts has been identified in the intestinal mucosa of CD patients. A significant loss in the bacteria that produce short-chain fatty acids (SCFAs) with anti-inflammatory properties, such as propionate, is also a consequence of dysbiosis in CD patients. Here, the AIEC reference strain LF82 was able to degrade propionate in the gut, which was sufficient to counteract the anti-inflammatory effect of propionate both in *in vitro* models and in mice with DSS-induced colitis. The consumption of propionate by AIEC pathobionts leads to an increase in TNF-α production by macrophages upon infection through the bacterial methyl-citrate pathway. To induce the protective effects of SCFAs on the inflamed gut, we used a G-protein-coupled receptor 43 agonist (GPR43 agonist) that is not metabolizable by intestinal bacteria. Interestingly, this agonist showed anti-inflammatory properties and decreased the severity of colitis in AIEC-infected mice, as assessed by an improvement in the disease activity index (DAI) and a decrease in AIEC pathobiont encroachment. Taken together, these results highlight the effectiveness of GPR43 agonist treatment in the control of gut inflammation and improved our understanding of the ability of AIEC to modulate propionate availability to create an infectious niche to its advantage.

## Introduction

The gut microbiota influences health and the nutritional stage *via* multiple mechanisms, and a large amount of evidence has shown that microbial metabolites have a major effect on host physiology. Short-chain fatty acids (SCFAs), predominantly acetate, propionate, and butyrate, are produced in the human large intestine by anaerobic fermentation of undigested carbohydrates, crude fibers, and polysaccharides.^1^ Depending on diet and gut microbiota composition, the intestinal SCFAs concentration can range from 60 to 150 mmol/L,^2^ with butyrate, propionate, and acetate present at a nearly constant molar ratio of 15:25:60.^3^ SCFAs have various beneficial roles in the gastrointestinal tract: they provide energy to the gut epithelium, promote intestinal epithelial integrity and are involved in the regulation of immune and inflammatory responses.^4–7^ Propionate has potent immunomodulatory effects and reduces the colonic expression of proinflammatory factors in mice with DSS-induced colitis.^8^ Most effects on intestinal inflammation are achieved through activation of the G protein-coupled receptor 43 (GPR43) SCFAs receptor,^9^ which is predominantly expressed in the colonic epithelium, adipose tissue and immune cells.^10–12^ Contradictory findings have been reported in GPR43^-/-^ mice regarding inflammatory phenotypes, leading to a lack of consensus.^13^ Confirming the role of this SCFAs receptor in human physiology is necessary to better understand the implications for intestinal inflammation, especially in the context of Crohn’s disease (CD). Recently, a fascinating study highlighted the role of Reg3-mucosal lectins, gut microbiota-derived propionate and its GPR43 receptor as a crucial mediator axis for gut epithelial regeneration in colitis.^14^

Acetate and propionate serve as the most potent activators of the GPR43 receptor, followed by butyrate and other SCFAs.^6^ In contrast to acetate, propionate levels have been reported to be markedly decreased in patients with CD compared with healthy individuals.^15^ Nevertheless, a recent study observed no significant differences in fecal concentrations of SCFAs in CD patients.^16^ Thus, these variations in SCFAs concentrations in CD patients could depend on the cohort and be the consequence of a specific treatment, such as exclusive enteral nutrition (EEN) or specific diet therapy.^17^

The abundance of propionate-producing bacterial species, such as *Veillonella* and *Bacteroides fragilis*, decreases in CD patients with low propionate levels.^15^ The decrease in the abundance of Bacteroidetes is a feature regularly found in CD patients and could contribute to inflammation^18^ since some bacteria belonging to this phylum, such as *Bacteroides fragilis*, have also been shown to exhibit protective effects in a mouse model of colitis.^19–21^ These results support the notion that the metabolism of propionate may play an important role in the pathogenesis of CD. It has also been consistently reported that CD patients have relatively high amounts of *Escherichia coli*, particularly the adherent-invasive *E. coli* (AIEC) pathobionts.^22–26^ These bacteria strongly adhere to and invade intestinal epithelial cells (IECs), survive within macrophages, move into deep tissues and activate immune cells, inducing inflammatory cytokines secretion.^27^ We have previously identified various pathways, including serine and ethanolamine catabolism and propionyl-CoA utilization, that may help in the metabolic adaptation of AIEC strains to their environments.^28,29^ In *E. coli*, propionate is converted to propionyl-CoA, which is a coenzyme A derivative of propionic acid, is transformed into 2-methyl-citrate by the methyl-citrate synthase (encoding by the *prpC* gene) and then into pyruvate and succinate. If the *prpC* gene is deleted, the methyl-citrate cycle will not occur. We found that genes involved in this pathway, such as *prpC*, were strongly upregulated in the AIEC strain LF82 under intestinal conditions (in the presence of bile and mucins).^28^ The aim of this study was to determine whether AIEC pathobionts are able to degrade propionate in the intestinal tract of CD patients and to evaluate the effect of propionate catabolism on host responses to AIEC infection.

## Results

### *The fecal abundance of* E. coli *in CD patients colonized by AIEC is inversely correlated with propionate concentration in the gut*

**Figure 1.**
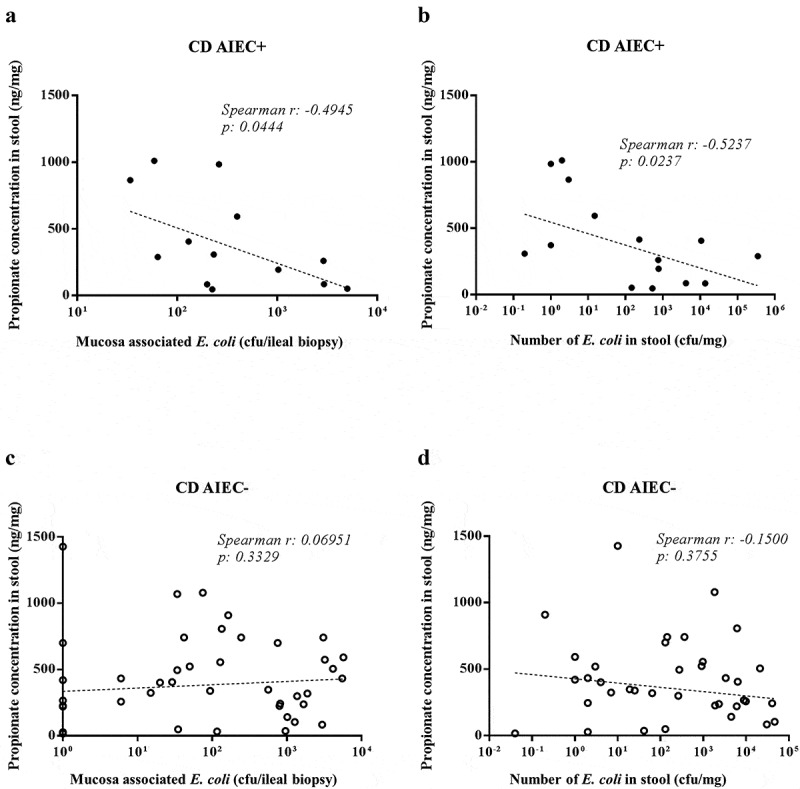
Inverse correlation between the fecal level of propionate and the number of *E. coli* in the stool samples and ileal mucosa of Crohn’s disease patients

A prospective and multicenter study including CD patients was conducted in our unit, and the abundance and global invasive ability of *E. coli* were analyzed in stool samples and in ileal specimens of CD patients colonized by ileal AIEC (CD AIEC+) or non-AIEC (CD AIEC-) strains. We quantified the acetate and propionate concentrations in stool samples from patients colonized or not colonized with AIEC bacteria. The acetate concentration in stools was negatively correlated with the fecal abundance of *E. coli* in CD AIEC+ (r = −0.6416, *p = 0.0058*; **Figure S1**), while no correlation was observed between the acetate concentration in stools and ileal abundance of *E. coli* (r = −0.3077, *p = 0.1531*). Interestingly, the propionate concentration in stools was negatively correlated with both the ileal and fecal abundances of *E. coli* for CD AIEC+ (r = −0.4945, *p = 0.0444*; r = −0.5237, *p = 0.0237*, respectively) but not for CD AIEC- (r = −0.06951, *p = 0.3329*; r = −0.1500, *p = 0.3755*) (Figure 1). This decreased level of propionate might be due to decreased abundance of propionate-producing bacteria and/or an increase in propionate degradation by the gut microbiota of AIEC-colonized CD patients and possibly by the AIEC themselves.

Correlation between the level of propionate and the number of *E. coli* bacteria associated with the ileal mucosa (a, c) and present in the stool samples (b, d) from Crohn’s disease patients; AIEC+ (a, b) and AIEC- (c, d). Spearman correlation analysis was performed between propionate concentration and the number of *E. coli* in pairwise comparisons (CFU: colony-forming unit).

### *The AIEC LF82 strain counteracts the anti-inflammatory effect of propionate* in vitro

**Figure 2.**
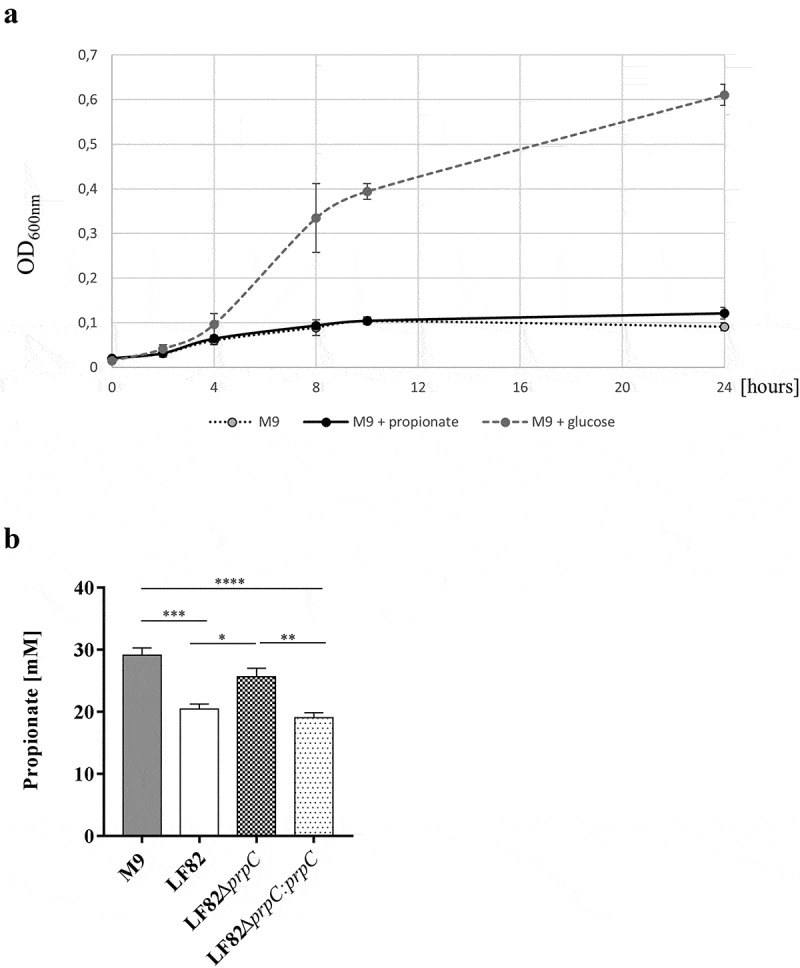
The AIEC strain LF82 catabolizes propionate

**Figure 3.**
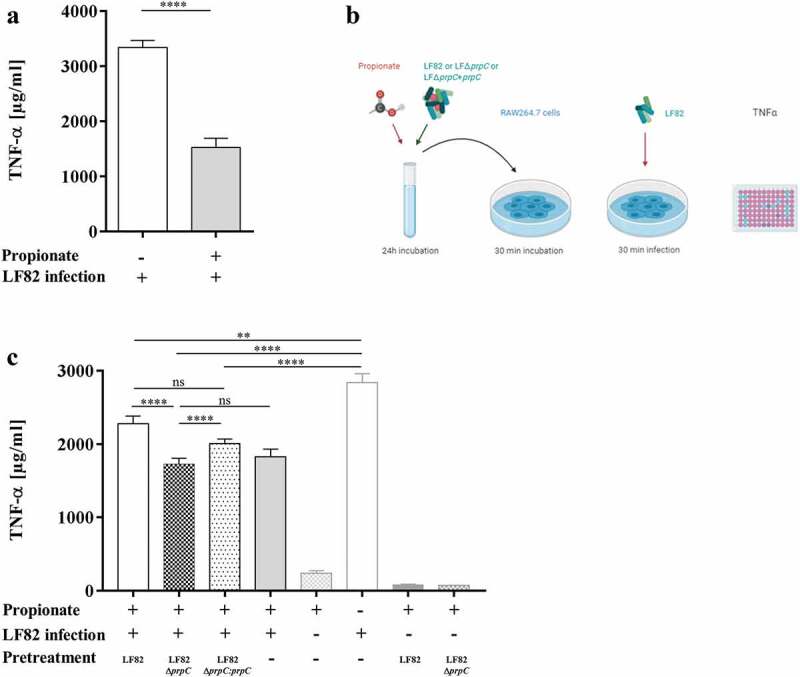
The AIEC LF82 strain counteracts the anti-inflammatory effect of propionate *in vitro.*

To evaluate the ability of the AIEC LF82 strain to utilize propionate as the sole source of carbon, propionate was added to a minimal medium. As shown in Figure 2a, the AIEC LF82 strain replicated extremely poorly in minimal medium (M9 or M9 containing propionate). To determine whether the AIEC strain LF82 catabolizes propionate, the AIEC strain LF82, the LF82Δ*prpC* isogenic mutant and the corresponding trans-complemented mutant LF82Δ*prpC:prpC* were grown in minimal medium supplemented with 30 mM propionate. After 24 h of incubation, the propionate concentration was significantly lower in medium incubated with the LF82 strain and the LF82Δ*prpC:prpC* mutant than in minimal medium incubated with the LF82Δ*prpC* mutant (Figure 2b). This result indicates that the AIEC strain LF82 is able to degrade propionate through the methyl-citrate pathway. It has been reported that propionate reduces the production of proinflammatory factors, including TNF-α.^30,31^ To determine whether the degradation of propionate by AIEC LF82 can have an impact on the production of proinflammatory cytokines, murine macrophage RAW264.7 cells were used. First, these cells were incubated with or without propionate for 30 min before a short period of infection with AIEC LF82 (MOI 10). As expected, propionate decreased the production of TNF-α by 54% (Figure 3a). Then, the AIEC LF82 strain, the LF82Δ*prpC* mutant and the trans-complemented LF82Δ*prpC:prpC* strain were preincubated with propionate for 24 h. A medium with propionate preincubated without addition of bacteria was used as control. The RAW264.7 cells were incubated with this conditioned medium, and then the cells were infected or not with the LF82 strain (Figure 3b).

(a) Bacterial growth curves of the LF82 strain in M9 supplemented with 30 mM propionate or 10 mM glucose. (b) The AIEC strain LF82, the AIEC LF82Δ*prpC* isogenic mutant and the corresponding trans-complemented mutant LF82Δ*prpC:prpC* were grown in minimal medium supplemented with 30 mM propionate. After 24 h of incubation, the propionate concentration was measured. The experiment was performed in duplicate with n = 3 at each time. The results are the mean ± SEM. Statistical comparisons were carried out by one-way ANOVA with the Bonferroni post hoc test (*p < 0.05, **p < 0.01) after normality testing using the Kolmogorov-Smirnov test.

(a) Secreted TNF-α amounts, quantified by ELISA, in supernatants of murine macrophage RAW264.7 cells incubated or not with propionate (30 mM) for 30 min before a short period of infection with AIEC LF82 (MOI 10). (b) The AIEC LF82 strain, the LF82Δ*prpC* mutant and trans-complemented LF82Δ*prpC:prpC* were preincubated with propionate for 24 h. The RAW264.7 cells were incubated with this incubation medium before infection with the LF82 strain. Secreted TNF-α levels in supernatants of murine RAW264.7 macrophages were measured. (c) Secreted TNF-α amounts in supernatants of murine macrophage RAW264.7 cells incubated with LF82, LF82Δ*prpC* or LF82Δ*prpC:prpC* (MOI 10) pretreated with propionate (propionate +) before incubation with cells and then infected or not with AIEC LF82 (n = 3). The results are the mean ± SEM of three experiments. Statistical analysis was carried out by normality testing using the Kolmogorov-Smirnov test and subsequent two-tailed Student’s t-test (A) or a one-way ANOVA with the Bonferroni post hoc test (c) (*p < .05, **p < .01, ****p < .0001).

TNF-α levels were significantly reduced in cells incubated with propionate pretreated with the mutant LF82Δ*prpC* compared to the levels in cells incubated with propionate pretreated with the LF82 or LF82Δ*prpC:prpC* strain (Figure 3c). In addition, no significant difference was observed between cells incubated with propionate pretreated with the mutant LF82Δ*prpC* and cells incubated with propionate but not pretreated with bacteria.

These results suggest that propionate degradation by AIEC pathobionts could participate in the uncontrolled production of proinflammatory mediators in AIEC-positive CD patients.

### *The AIEC LF82 strain decreases the propionate concentration* in vivo

**Figure 4.**
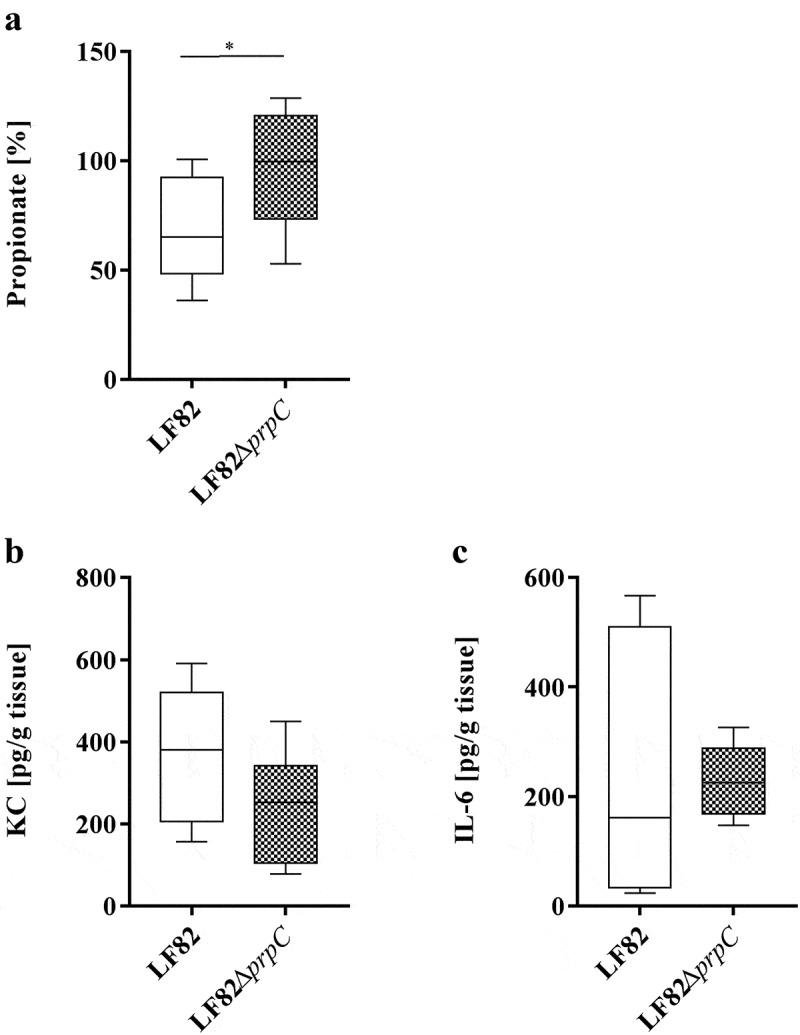
AIEC LF82 decreases the propionate concentration *in vivo.*

CEABAC10 mice were orally challenged with the AIEC LF82 strain or the LF82Δ*prpC* isogenic mutant. Quantification of bacteria in stool samples or of bacteria associated with the intestinal mucosa showed no difference in colonization between the wild-type and mutant strains, suggesting that propionate metabolism is not essential for AIEC LF82 colonization (**Figure S2**). The fecal samples of each mouse were pooled before and after infection. The mean concentration of propionate before infection was normalized as 100% (95 ± 19 or 80 ± 22 µg/g fecal propionate in mice infected with LF82 or LF82Δ*prpC*, respectively). The results showed a 40% decrease in propionate concentration in mice infected with the AIEC LF82 strain (63 ± 18 µg/g propionate), while no overall decrease was observed in mice infected with LF82Δ*prpC* (73 ± 17 µg/g propionate, Figure 4a). The decrease in the propionate concentration was not associated with increased secretion of the proinflammatory chemokine KC (keratinocyte-derived chemokine, Figure 4b) or cytokine IL-6 (Figure 4c). However, it has been previously described that AIEC requires a particular context to participate in the inflammatory process, such as colitis or antibiotic treatment.^32^

CEABAC10 mice (n = 8 for each group) were pretreated with fosfomycin (2 g/L) for 4 days and orally infected with the LF82 strain or the isogenic mutant LF82Δ*prpC*. (a) Propionate concentrations in fecal samples of mice collected after infection with LF82 or LF82Δ*prpC*. For each mouse, fecal samples collected before infection with the LF82 strain were pooled, and the concentration of propionate was determined as 100%. (b) Secreted KC chemokine and (c) IL-6 cytokine amounts from colonic tissue of infected mice 2 days post infection. The results are presented as the median. Statistical comparisons were carried out by normality testing using Kolmogorov-Smirnov tests and a subsequent two-tailed Student’s t-test (*p < .05).

### The AIEC LF82 strain counteracts the anti-inflammatory effect of propionate in mice

**Figure 5.**
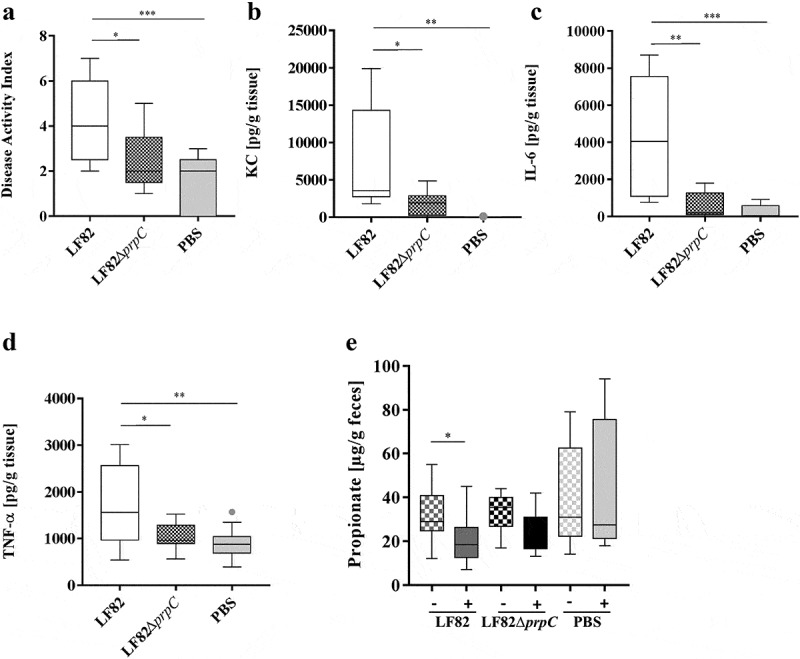
The AIEC LF82 strain counteracts the anti-inflammatory effect of propionate in mice

Pathobiont AIEC LF82 bacteria exacerbate colitis in DSS-treated mice,^33^ and it has been reported that oral administration of propionate could ameliorate DSS-induced colitis mainly by reducing inflammation.^8^ Mice treated with DSS and supplemented with propionate were infected with LF82 strain. The effect of propionate supplementation on colonic inflammation was evaluated by determining the disease activity index (DAI) score and by detecting the release of proinflammatory cytokines by colonic tissues. No significant difference in disease activity index and the release of KC and IL-6 was observed between mice treated or not with propionate. No statistically significant difference of ileal colonization by LF82 was also observed after propionate supplementation (**Figure S3**). The lack of a significant anti-inflammatory effect of propionate may be related to its degradation by AIEC bacteria. To test this hypothesis, mice treated with DSS and supplemented with propionate were infected with LF82 or the LF82Δ*prpC* mutant. The DAI score of mice infected with AIEC LF82 bacteria was significantly higher than that of mice infected with the LF82Δ*prpC* mutant or uninfected mice (control mice, Figure 5a). The released levels of the proinflammatory chemokine KC (keratinocyte-derived chemokine), cytokines IL-6 and TNF-α were very low in the control mice (Figure 5b–Figure 5d), probably because propionate decreased DSS-induced inflammation. In contrast, the levels of these cytokines were increased in mice infected with *E. coli* strains, and they were significantly higher in mice infected with LF82 bacteria than in mice infected with LF82Δ*prpC*. These results reveal that the LF82 strain can counteract the anti-inflammatory effect of propionate in transgenic mice and suggest that the methyl-citrate pathway is involved in the virulence of the AIEC LF82 strain. Quantification of bacteria in stool samples or associated with the intestinal mucosa showed no difference in colonization between the wild-type and mutant strains (**Figure S4**). To determine whether inflammation was associated with a decrease in propionate levels in the mouse gut, fresh fecal samples were collected before and after infection for each mouse. The propionate concentration was significantly decreased in mice infected with the LF82 strain compared to the basal level (*p* = 0.045), in contrast to the level in mice infected with the LF82Δ*prpC* mutant (*p* > .999) (Figure 5e). In the control group, this propionate concentration did not vary (*p* > .999). Overall, these results suggest that AIEC pathobionts may be able to decrease the propionate concentration in the intestine, which would impair the control of inflammation by this SCFA.

CEABAC10 mice (n = 9, two independent experiments pooled) were pretreated with an antibiotic cocktail containing 500 mg/L metronidazole, 1 g/L streptomycin, 1 g/L neomycin and 1 g/L ampicillin. Mice received 1% DSS and, when needed, 0.6% calcium propionate in their drinking water in parallel with antibiotic treatment. On day 5, the antibiotic treatment was stopped; 0.25% DSS and 0.6% propionate were maintained in the drinking water. On day 6, mice were orally infected with AIEC LF82, the isogenic mutant LF82Δ*prpC*, or PBS as a control. (a) Disease activity index of mice. (b–d) Secreted KC chemokine, IL-6 and TNF-α cytokines amounts from colonic tissue of mice. (E) Propionate concentrations from fecal samples of mice before and after infection. The results are presented as the median values. Statistical comparisons were carried out by normality testing using Kolmogorov-Smirnov tests, and a subsequent one-way ANOVA with Bonferroni post hoc test was performed (a–d). A nonparametric Friedman test was applied to assess differences in propionate concentration before and after infection (e) (*p < .05, **p < .01, ***p < .001).

### The GPR43 agonist decreases the severity of colitis in AIEC LF82-infected mice

**Figure 6.**
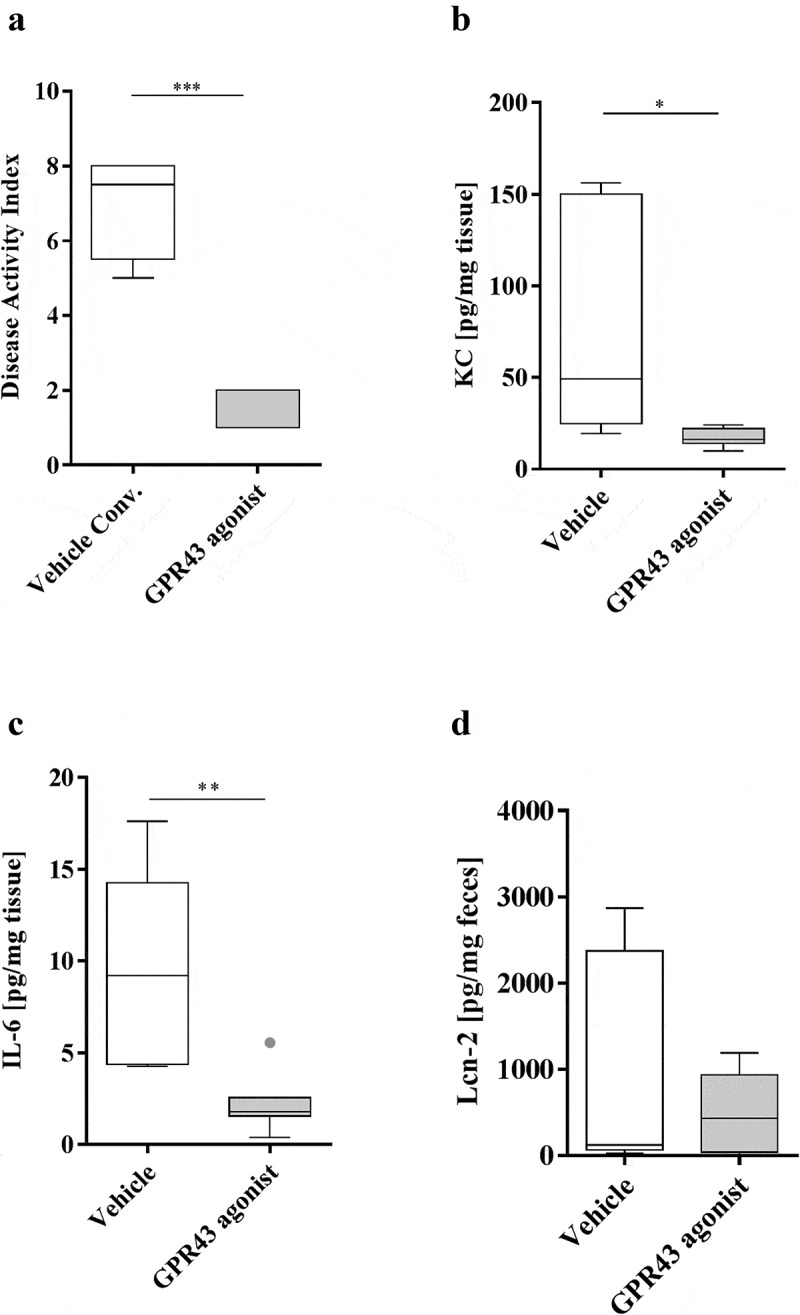
The GPR43 agonist decreases the severity of colitis in AIEC LF82-infected mice

**Figure 7.**
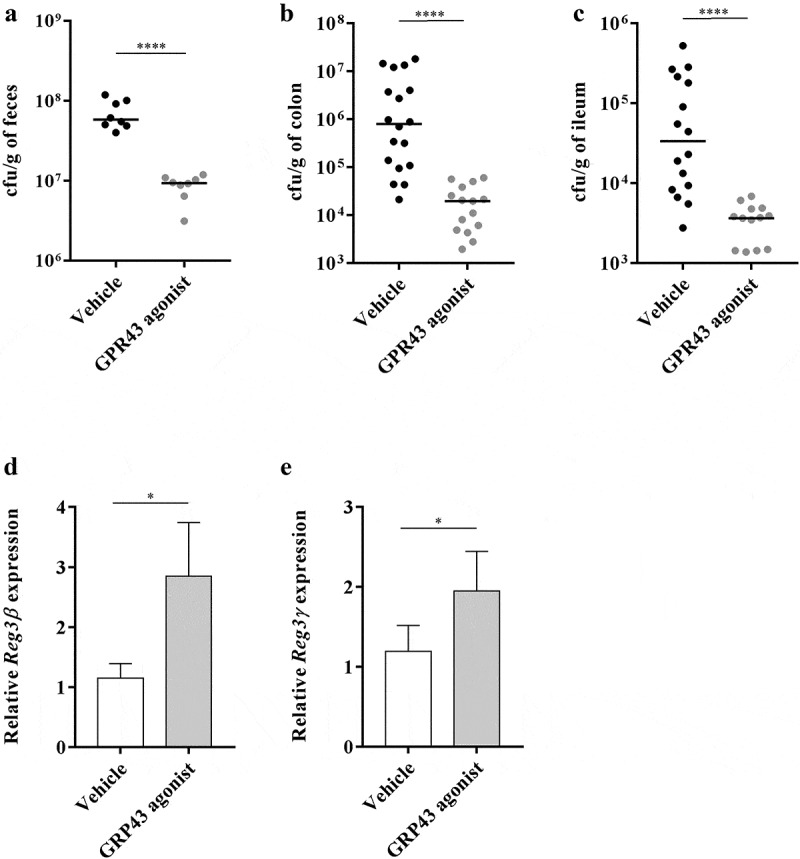
The GPR43 agonist displays anti-adhesive properties in AIEC LF82-infected mice

The anti-inflammatory effect of propionate is mediated by activation of the GPR43 receptor. We tested the efficiency of an agonist of the GPR43 receptor that cannot be metabolized by intestinal bacteria to control the inflammation induced by the AIEC pathobiont. Thus, we evaluated the impact of GPR43 agonist administration on gut inflammation in AIEC LF82-infected mice. The DAI score indicated that the GPR43 agonist was effective in decreasing the signs of colitis induced by AIEC colonization from day 10 post infection (Figure 6a). The release of the proinflammatory cytokines KC and IL-6 in mice treated with the GPR43 agonist was significantly lower than that in mice receiving the vehicle only (5.5- and 4.3-fold decrease, respectively, Figure 6b,Figure 6c). Moreover, the GPR43 agonist led to decreased fecal levels of lipocalin-2, which is a sensitive biomarker for intestinal inflammation (Figure 6d). Thus, we demonstrated the anti-inflammatory effect of GPR43 receptor agonists in the context of AIEC encroachment. In addition, the GPR43 agonist seems to be very effective in eliminating AIEC bacteria from the gut, as observed by quantification in the fecal samples and in the intestinal tissues. Indeed, 2-days post infection, a significant decrease in the AIEC LF82 bacterial load in feces was observed for mice treated with the GPR43 agonist compared to mice receiving the vehicle only (Figure 7a). In addition, in mice treated with the GPR43 agonist, the number of bacteria associated with colonic and ileal tissues was significantly reduced (Figure 7b,Figure 6c). These results demonstrate that GPR43 agonist treatment effectively decreases AIEC LF82 colonization in the gut of transgenic mice. Propionate treatment has been shown to upregulate the expression of Reg3β and -γ, which are antimicrobial peptides at mucosal surfaces of the gut, in the colon of DSS-treated mice.^14^ Therefore, we analyzed Reg3 mRNA expression in mice treated or not with the GPR43 agonist and observed a significant increase of Reg3β and Reg3γ levels in mice treated with the GPR43 agonist compared to untreated mice (Figure 7d,Figure 6e). These observations reinforce the hypothesis that GPR43 agonist supplementation could participate in the protection of mice against AIEC LF82 bacterial infection. Interestingly, Park *et al*. demonstrated that SCFAs could restore the turnover of IECs in antibiotic-treated mice.^34^ In our study, the GPR43 agonist treatment does not modify the colonic expression level of genes implicated in epithelial differentiation (*Olfm4, Hes1, Atoh1* and *Muc2*) and IEC proliferation (*Pcna, CyclinD1* and *CyclinA*). Additionally, we did not observe any change in expression of colonic crypt length measurements, suggesting that the protection mediated by the GPR43 agonist seems to be more targeted toward an anti-inflammatory phenotype (**Figure S5**).

CEABAC10 mice (n = 8 for each group) were pretreated with an antibiotic cocktail containing 500 mg/L metronidazole, 1 g/L streptomycin, 1 g/L neomycin and 1 g/L ampicillin. Mice were orally challenged for 7 days with 10^9^ CFU of AIEC LF82 bacteria and with 5 mg/kg/day GPR43 agonist. Simultaneously, the drinking water of the mice was supplemented with 0.25% DSS. (a) Disease activity index (DAI) of AIEC LF82-exposed mice on day 10. (b,c) Secreted KC and IL-6 cytokines in colonic tissue culture supernatant. (d) Secreted lipocalin-2 (Lcn-2) in the feces of mice treated with the GPR43 agonist and of untreated mice. The results are presented as the median values. Statistical comparisons were carried out by normality testing using Kolmogorov-Smirnov tests, and a subsequent two-tailed Student’s test (b,c) or Mann–Whitney U-test (a–d) was performed (*p < .05, **p < .01, ***p < .001).

CEABAC10 mice were infected with AIEC LF82 and treated orally by gavage with 5 mg/kg/d GPR43 agonist or vehicle only. (a) AIEC LF82 quantification in the feces of mice treated with the GPR43 agonist compared to mice treated with vehicle 2 days post infection (n = 8 for each group). (b,c) Colonic and ileum-associated *E. coli* bacteria from infected CEABAC10 mice 2 days post infection (n = 23, three independent experiments pooled). (d,e) Reg3β and Reg3γ mRNA were quantified by RT-qPCR in colonic mucosa of mice (n = 8 for each group). Each symbol represents an individual mouse, and lines show medians (a–c). The results of mRNA expression are the mean ± SEM (d,e). Statistical comparisons were carried out by normality testing using Kolmogorov-Smirnov tests, and a subsequent two-tailed Student’s test (a) or Mann–Whitney U-test (b,c) or non-parametric one-tailed Mann-Whitney U-test (D-E) was performed (*p < .05, ****p < .0001).

## Discussion

In the present study, we showed that the AIEC LF82 strain counteracts the anti-inflammatory effect of propionate in a mouse model of DSS-induced colitis. First, we found that propionate reduced inflammation in the colonic tissues of mice, which is consistent with other studies.^8,35^ Oral administration of propionate has beneficial effects on the intestinal epithelium by improving intestinal barrier function, inhibiting inflammation, and modulating oxidative stress in mice with DSS-induced colitis.^8^ Propionate also regulates the size and function of the colonic Treg pool and protects against colitis in a GPR43-dependent manner in mice.^35^ AIEC exacerbate intestinal inflammation in mice treated with DSS.^36^ In the present study, we revealed that colonic inflammation induced by AIEC pathobionts in transgenic mice is not prevented by propionate administration but is prevented by a nonmetabolizable GPR43 receptor agonist. We showed here that AIEC bacteria are able to degrade propionate *in vitro*. In contrast, a mutant with a nonfunctional *prpC* gene cannot catabolize propionate through the methyl-citrate pathway. This AIECΔ*prpC* mutant failed to induce an inflammatory response in mice. These results suggest that AIEC pathobionts counteract the anti-inflammatory effect of propionate in mice by degrading this compound. This hypothesis is reinforced by the decreased propionate concentration in fecal samples in mice infected with AIEC pathobionts. Additionally, we found a negative correlation between the propionate concentration and the fecal abundance of *E. coli* in stool samples of CD patients colonized by AIEC pathobiont strains. Our results thus support the idea that a high rate of colonization by AIEC pathobionts causes a decrease in propionate concentration, which may be associated with impaired regulation of intestinal inflammation in CD patients.^15,37^ However, the high abundance of *E. coli* in the gut of CD patients could also be associated with decreased SCFAs production since butyrate-producing bacterial species, such as *Faecalibacterium*, and propionate-producing bacterial species, such as *Veillonella* and *Bacteroides fragilis*, are decreased in CD patients. The decrease in the abundance of *Faecalibacterium* and *Bacteroidetes* is a feature regularly found in CD patients.^36^ Hence, it could conceivably be hypothesized that the decrease in propionate observed in CD patients is due to the low abundance of propionate-producing bacteria combined with the high abundance of *E. coli* strains such as AIEC pathobionts that are able to degrade propionate.

Prior studies have noted the importance of SCFAs for constant repair of the intestinal epithelium, suggesting that propionic and butyric acid could be useful in the treatment of inflammatory disorders, including CD.^38,39^ However, contradictory effects of SCFAs on intestinal inflammation in patients with colitis and in murine models have been reported.^40–42^ The variable abundance of propionate-degrading bacteria, such as AIEC, could explain these inconsistent effects to a certain extent. It has recently been shown that AIEC pathobionts adapt to prolonged exposure to propionate, resulting in an increase in virulence. Indeed, upon exposure to propionic acid, highly virulent AIEC variants having enhanced AIEC phenotype emerge (increase in adhesion, invasion and biofilm formation).^43^ We completed this data analysis to reveal that AIEC degrade propionate through the methyl-citrate pathway and counteract the anti-inflammatory effect of propionate. We have previously shown that in the presence of bile, an interaction among ethanolamine utilization, 1,2 propanediol degradation and the methyl-citrate pathway provides an energetic advantage to the AIEC LF82 strain.^28^ A study by Ormsby et al.^44^ reinforced the link between these pathways by showing that propionate stimulates AIEC-mediated degradation of ethanolamine. Additionally, ethanolamine utilization confers a competitive advantage to AIEC strains in gut colonization.^28^ Intestinal ethanolamine is readily available during periods of intestinal inflammation.^44^ In summary, it can be suggested that dietary supplementation with propionate may be inefficient, if not harmful, for CD patients colonized by AIEC bacteria.

Thus, one of the possible strategies to restore the beneficial effects of SCFAs or fermentable dietary fibers would be to use nonmetabolizable SCFAs-G-protein-coupled receptor agonists. In the present study, we evaluated the efficacy of a G-protein-coupled receptor 43 agonist against gut inflammation induced by AIEC pathobiont infection in a mouse model and showed that the GPR43 receptor agonist significantly decreased the severity of colitis in the presence of AIEC pathobionts in the gut. It has been reported that activation of the GPR43 receptor has a protective effect against colitis by favoring the differentiation and function of colonic Tregs.^35,45,46^ One unanticipated finding was that GPR43 agonist administration to mice counteracted the intestinal colonization by AIEC pathobionts. This result may be explained by the overall decrease in inflammation. Indeed, although it is recognized that AIEC pathobionts may be considered an initiating factor for inflammation in CD, it has been previously reported that persistent colonization by AIEC pathobionts is also favored in an inflammatory context,^32,47^ and overgrowth of the AIEC population may be related to a metabolic shift to catabolize L-serine in the inflamed gut.^29^ Moreover, activation of the GPR43 receptor is associated with neutrophil chemotaxis, T cell differentiation, activation and subsequent cytokines production.^6^ The role of the SCFAs/GPR43 axis was demonstrated in the maintenance of epithelial integrity, inducing mucosal healing and suppressing inflammation.^7^ In a previous study, we demonstrated that downregulation of SCFAs-sensitive GPR43 is associated with gut inflammation, a phenomenon restored by GPR43 agonist treatment of DSS-induced colitis.^48^ Recently, propionate induces Reg3 in intestinal organoids and in gnotobiotic mice colonized with a defined microbiota-producing SCFAs.^14^ Reg3β has bactericidal activity against Gram-negative bacteria. Moreover, Park *et al*. demonstrate that Gram-positive commensal bacteria are a major determinant of IEC turnover, and that their stimulatory effect seems to be mediated by SCFAs.^34^ In our study, the GPR43 agonist seems to induce Reg3β and Reg3γ genes expression that probably could contribute to the decrease of AIEC LF82 colonization. No induction of epithelial differentiation and proliferation mechanisms was observed, that could suggest that the effects of the GPR43 agonist are more targeted toward an anti-inflammatory phenotype.

Interestingly, we demonstrate here the efficiency of this GPR43 agonist in AIEC LF82-infected transgenic CEABAC10 mice. Therefore, as GPR43 regulates the colonization and/or encroachment abilities of the AIEC pathobiont, it could be relevant as a therapeutic approach for AIEC-colonized CD patients.

In summary, our study identified a new mechanism that improves our understanding of the proinflammatory potential of AIEC pathobionts in the context of CD. Indeed, AIEC pathobiont bacteria seem to be able to impair the anti-inflammatory effects of propionate by creating an infectious niche, leading to gut inflammation. Importantly, these results are relevant to humans since low propionate concentrations were also reported in CD patients colonized by AIEC pathobionts. In addition, by providing key evidence of the importance of SCFAs in intestinal protection, this work lays the groundwork for further studies aiming to correct SCFAs deficiency in CD patients by GPR43 agonist supplementation. In conclusion, our data provide insights for new preventive or curative treatments for CD.

## Methods

### Mice and ethics statement

Mice were maintained under specific-pathogen-free conditions (21–22°C, 12:12-h light-dark cycle) in the animal care facility of the University Clermont Auvergne (Clermont-Ferrand, France). C57BL/6 female mice were purchased from Charles River Laboratories France for reproduction with heterozygotic transgenic CEABAC10 males^49^. Littermates of the >10^th^ backcross were used for experimentation. All transgenic CEABAC10 mice used in this study were 5 to 6 weeks old. Animal experiments were performed according to the institutional guidelines approved by the CEMEA Auvergne committee for ethical issues (00730.02).

### Bacterial strain and media

The *E. coli* AIEC strain LF82 was isolated from a chronic ileal lesion of a patient with CD and belongs to *E. coli* serotype O83:H1.^50^ *E. coli* LF82 was grown overnight at 37°C in Lysogeny Broth (LB). Amoxicillin (32 mg/L), kanamycin (50 mg/L) and chloramphenicol (25 mg/L) (Sigma-Aldrich) were added when required. The ability of *E. coli* LF82 to grow with propionate (calcium propionate, Sigma-Aldrich) was tested on minimal medium M9 containing Na_2_HPO_4_ (48 mM), KH_2_PO_4_ (22 mM), NH_4_Cl (20 mM), MgSO_4_ (1 mM), CaCl_2_ (0.1 mM), vitamin B12 (cyanocobalamin) (150 nM), vitamin B1 (5 mg/L), and trace metals (0.1 µM ZnSO_4_, 0.045 µM FeSO_4_, 0.2 µM Na_2_Se_2_O_3_, 0.2 µM Na_2_MoO_4_, 2 µM MnSO_4_, 0.1 µM CuSO_4_, 3 µM CoCl_2_ and 0.1 µM NiSO_4_) (2 mL/L). To study the ability of *E. coli* LF82 to degrade propionate (calcium propionate, Sigma-Aldrich), a pre-culture was performed by growing *E. coli* in M9 medium supplemented with 20% LB and 1% bile salts. Cells taken from the pre-culture (10^8^ cells per ml) were incubated on minimal medium supplemented with 30 mM propionate and 10 mM glucose (0.025%).

### Construction and transcomplementation of isogenic mutants

*E. coli* LF82 was transformed with pKOBEG, a plasmid encoding the Red proteins that protect linear DNA from degradation in bacteria. The plasmid was maintained in bacteria at 30°C with 25 mg/L chloramphenicol and 1 mM L-arabinose. The Flp recognition target-flanked cassette harboring the kanamycin resistance cassette was generated by PCR from *E. coli* BW25141 with d-prpC-F/d-prpC-R primers (**Table S1**) and high-fidelity Platinum Taq polymerase (Invitrogen) according to the manufacturer’s instructions. The PCR products were electroporated in *E. coli* LF82 cells previously washed with glycerol. The resulting LF82Δ*prpC* isogenic mutant (Km^R^) was selected on Mueller-Hinton agar containing 50 mg/L kanamycin. The replacement of the *prpC* gene by the kanamycin resistance cassette was confirmed by PCR (**Table S1**). The kanamycin resistance cassette was then removed from LF82Δ*prpC* bacteria by transient expression of the Flp recombinase from the pCP20 plasmid, creating the LF82Δ*prpC* (Km^S^) strain.

The *prpC* gene was amplified by PCR from *E. coli* LF82 genomic DNA by using the NdeIprpC-F and EcoRIprpC-R primers (**Table S1**). The amplified DNA was purified with a NucleoSpin extraction kit (Macherey-Nagel), digested with NdeI and EcoRI (New England Biolabs), and ligated to the NdeI-EcoRI-digested expression vector pBK-CMV (Agilent Technologies). This construct was electroporated into LF82Δ*prpC* (Km^S^) electrocompetent strain and selected on Mueller-Hinton agar containing 50 mg/L kanamycin. The presence of the *prpC* gene was confirmed by PCR (LF82Δ*prpC:prpc*). The construct was checked by double-stranded DNA sequencing (GATC biotech, Germany).

### Mouse infection

Several experiments were performed in this study. First, sixteen C57BL/6 transgenic CEABAC10 mice (body weight ≈19–24 g) were pretreated by oral administration of fosfomycin in drinking water (2 g/L) for four days to eliminate commensal *E. coli*. Seven days after stopping antibiotic treatment, the animals were orally challenged with 10^9^ LF82 or LF82Δ*prpC* cells for four consecutive days. For experimentation with dextran sodium sulfate-induced colitis, transgenic CEABAC10 mice were provided drinking water with an antibiotic cocktail containing 500 mg/L metronidazole (Sigma-Aldrich), 1 g/L streptomycin (Euromedex), 1 g/L neomycin (Sigma-Aldrich), and 1 g/L ampicillin (Euromedex) for four days to disrupt the normal resident bacterial microbiota in the intestinal tract and to favor the implantation of the LF82 or LF82Δ*prpC* strain. The mice received 1% (wt/vol) DSS (molecular mass, 36,000 to 50,000 Da; MP Biomedicals) and, when needed, 0.6% calcium propionate (Sigma-Aldrich) in their drinking water together with the antibiotics. On day 5, the antibiotic treatment was stopped; 0.25% DSS and 0.6% propionate were maintained in the drinking water. On day 6, the mice were orally challenged with 10^9^ CFUs of the LF82 or LF82Δ*prpC* strain or with PBS for four consecutive days.

For GPR43 agonist administration, transgenic CEABAC10 mice received the same antibiotic cocktail in drinking water for 7 days, as described previously. The mice were then continuously exposed to 0.25% (wt/vol) DSS (molecular mass, 36,000 to 50,000 Da; MP Biomedicals) and simultaneously orally infected for 7 days with 10^9^ CFUs of AIEC LF82 bacteria and with 5 mg/kg/day GPR43 agonist (Calbiochem, Millipore) dissolved in 50% (1:2) dimethyl sulfoxide (DMSO) or vehicle (50% DMSO).

Mice were weighed daily. Fresh fecal samples (100–200 mg) were collected from individual mice 2 days before and after gavage for determination of propionate level. Fecal samples were collected 2-days post gavage, suspended in phosphate-buffered saline (PBS; Gibco) and plated onto Lysogeny Broth (LB; Conda) agar plates containing ampicillin (100 µg/mL; Euromedex) to isolate LF82 bacteria or on LB agar plates containing 50 µg/mL kanamycin (Euromedex) to isolate LF82Δ*prpC* and incubated at 37°C overnight. Antibiotic susceptibility testing was performed on bacteria in a random manner to confirm whether the isolates were LF82 or LF82Δ*prpC*. Two days post infection, mice were euthanized by cervical dislocation. To quantify AIEC bacteria associated with colonic and ileal tissues, intestinal tissues were cut longitudinally, washed in PBS, homogenized in 1 mL of PBS, and plated onto LB agar plates containing 100 µg/mL ampicillin or 50 µg/mL kanamycin. The severity of colitis was assessed by the disease activity index (DAI) score, which ranges from 0 (healthy) to 12 (high colitic activity) **(Table S2)**.^33^

### Cell culture

The murine macrophage RAW264.7 cells were maintained at 37°C in a humidified atmosphere of 5% CO_2_ in DMEM supplemented with 100 U/mL penicillin, 100 µg/mL streptomycin, and 10% fetal bovine serum (DMEMS). For experimental purposes, the cells were harvested in log phase and plated at a density of 5 × 10^5^ cells/mL of medium in a 24-well sterile plate. Then, the cells were pretreated for 30 min with incubation medium that was previously filtered (0.45 then 0.22 µm) before infection with the LF82 strain (MOI 10).^51^ After a 30-min incubation period at 37°C with 5% CO_2_, the medium was replaced with 1 mL of DMEMS medium containing 16 µg/mL ciprofloxacin. The next day, cytokines were quantified in the supernatant using an ELISA kit from R&D Systems according to the manufacturer’s instructions.

### Quantification of proinflammatory cytokine release

Intestinal tissues (1 cm) were placed in RPMI (Gibco) supplemented with antibiotics (50 µg/mL gentamicin (Euromedex) and 1% antibiotic and antimycotic solution X-100 (10,000 U of penicillin, 10 mg of streptomycin and 0.25 mg of amphotericin B per milliliter, PAA)) overnight in a 24-well culture plate in an atmosphere containing 5% CO_2_ at 37°C. All collected supernatants were filtered at 0.22 µm and frozen at −80°C until processing. Proinflammatory mouse cytokines released (IL-6 and KC) were quantified in the filtered culture supernatant by ELISA using kits from R&D systems following the manufacturer’s instructions.

### Quantification of fecal lipocalin-2 (Lcn-2)

Frozen fecal samples were reconstituted in PBS containing 0.1% Tween 20 (100 mg/mL; Euromedex) and vortexed to obtain a homogenous fecal suspension. These samples were centrifuged for 5 min at 10000 × g and 4°C. Supernatants were collected and stored at −80°C until analysis. ELISAs were performed by using DuoSet® ELISA Development Systems for Lcn-2 from R&D Systems according to the manufacturer’s instructions.

### Microbiological analyses of ileal biopsies and stool samples of CD patients

Stool samples of CD patients were obtained from a prospective multicenter study. This study was performed in accordance with the Declaration of Helsinki, good clinical practice guidelines and applicable regulatory requirements. The study was approved by the French ethical committee, the so-called *“Comité de Protection des Personnes (CPP) Sud-Est 6”* – France [AU 904]. In this prospective multicenter study (8 centers), all patients required ileocolonoscopy, regardless of the indication, and were consecutively included between September 2015 and September 2016. In addition to patient characteristics, clinical and endoscopic data were gathered **(Table S3)**. Stool samples of CD patients were collected on the day of colonoscopy. Biopsies were taken from the ileum of the patients, focusing on either macroscopically normal or ulcerated areas.

#### Determination of total E. coli number associated with ileal mucosa and in stools

Ileal biopsies were washed in phosphate-buffered saline (PBS), crushed (Ultra-Turrax, IKA) and incubated for 15 minutes on a tube rotator at room temperature in the presence of Triton 0.1X. Ten-fold dilutions of the lysate were then plated on Drigalski agar to number total *E. coli* colonies after 24 hours of incubation at 37°C. Results are given in colony-forming unit (cfu)/ileal biopsy. Ileal biopsies were carried out using calibrated biopsy forceps to obtain 94 mg of tissue and the number of *E. coli* cfu was determined on the whole biopsy. The stool samples, stored at −80°C in 15% glycerol Minimum Essential Medium (MEM), were crushed in physiological water. Ten-fold dilutions of the homogenate were then plated on Drigalski agar to number total *E. coli* colonies after 24 hours of incubation at 37°C. Results are given in colony-forming unit (cfu)/mg. A random selection of *E. coli* strains was performed on Drigalski plate followed by *E. coli* identification by mass spectrometry.

#### Phenotypical assays to identify AIEC bacteria

The AIEC characterization was carried out by analyzing their abilities to adhere to and invade intestinal epithelial cell lines, as well as survive and replicate within macrophage cell lines, by conducting gentamicin protection assays with intestine-407 epithelial cells (ATCC, CCL-6) and THP-1 macrophages (ATCC, TIB-202), as previously described.^23^

### Extraction of propionate from fecal samples

Mouse fecal samples were frozen at −20°C immediately after collection. For each mouse, fecal samples collected over 2 days before or after gavage with *E. coli* strains were pooled. Stool samples of CD patients from the previously described prospective multicenter study were used. Human and mouse samples were weighed and suspended in 1 mL of water with 0.5% phosphoric acid per 0.5 g of sample. Fecal suspensions were homogenized with vortexing for approximately 2 min and centrifuged for 10 min at 10000 × g.

The extraction procedure for short-chain fatty acids was adapted from the method previously been validated by Hoving et al.^52^ and García-Villalba et al.^53^ Simultaneous analyses of acetate and propionate were prepared by spiking 125 µL of internal standard (4-methylvaleric acid (MVA) at 200 mg/L) into 300 µL of biological sample. Derivatization of short-chain fatty acids was performed with 2,3,4,5,6-pentafluorobenzylbromide (PFBBr) at pH 6.8 with phosphate buffer for 1 hour at 60°C. Then, sample solutions were extracted with 200 µL of hexane. The upper organic layer was collected and transferred directly into chromatography vials for injection prior to analysis by gas chromatography.

### Quantification of propionate by gas chromatography and mass spectrometry

Analyses were performed on an HP5973 MS with an HP6890 series GC (Agilent Technologies, Atlanta, GA, USA). Automatic injections were performed using an HP6890 autosampler. The temperatures of the injector and the transfer line detector were 180°C and 280°C, respectively. The GC was operated in splitless injection mode with a constant flow of 1 ml/min of helium through the HP-5 MS column (30 m × 0.25 mm i.d. with 0.25 µm film thickness (J&W, Folsom, CA)). The GC oven temperature was programmed to start at 70°C, increasing first to 150°C at 20°C/min and then to 290°C at 30°C/min. The retention times were 3.39 min, 3.93 min and 5.35 min for acetate, propionate and MVA, respectively. Ions were detected by selective ion monitoring (SIM) for quantification (Q) and confirmation (q): m/z 240 (Q), 197 and 181 (q) for acetate; m/z 254 (Q), 197 and 181 (q) for propionate; and m/z 97 (Q), 57 and 115 (q) for MVA. Identification of the target compound was carried out by comparing the retention time and m/z ratio with those of the standards. HP Chemstation software was used to control the equipment and carry out the data processing. The concentrations of acetate and propionate in the biological samples were determined based on their area ratios to that of the IS using a weighted quadratic fit. The lower limit of quantification (LLOQ) for each compound was 5 mg/L, and the upper limit of quantification (ULOQ) was 1000 mg/L in biological samples without dilution.

### RNA-extraction and RT-qPCR

Total RNA from CEABAC10 colonic mucosa was extracted using Trizol reagent following the manufacturer’s instructions. Briefly, 1 cm of colonic mucosa was homogenized in liquid nitrogen using mortar and pestle. The resulting powder was suspended in 1 ml Trizol reagent (Life Technologies) and 200 µL of chloroform were added. The tubes were vortexed and spin at 12,000 g for 10 min at 4°C. The aqueous phase was transferred into a new tube and 500 µL of isopropanol were added for 30 min at RT for RNA precipitation. The tubes were spun at 12,000 g for 10 min at 4°C and the pellet containing RNA was washed twice with 70% ethanol. The pellet was suspended in 50 µL RNase-free water. The RNA quality was assessed by bioanalyzer and their concentration was determined by fluorimeter Qubit 2.0 (Thermo Fisher Scientific) for RT-qPCR or mRNA-sequencing. mRNA were reverse transcribed using PrimeScript RT Reagent kit (Takara) following the manufacturer’s instructions. After cDNA dilution (1/10), 1 µL of cDNA was used as a template for qPCR quantification (iTaq Universal SYBR Green Supermix, Bio-Rad). *Hprt* gene (hypoxanthine phosphoribosyltransferase) was used as an endogenous control to normalize the target gene expression. For analysis, the fold change for the target gene was calculated using the 2− ΔΔCT method after normalization to controls. Specific primer sequences used are listed in Supplementary Table S1 and each primer pair was designed on two different exons to span a large intronic region.

### Statistical analysis

Statistical analyses were performed using the GraphPad Prism V.7.0 (GraphPad Software, San Diego, CA, USA) software package for PC. For all data displayed in graphs, values are expressed as the mean ± SEM or median. Data comparisons between two groups were performed using a two-tailed Student’s t-test analysis or a Mann–Whitney U-test depending on the normality test using the Kolmogorov–Smirnov test. A non-parametric Friedman test was applied to assess differences in propionate concentration before and after infection. When appropriate, a one-way ANOVA with the Bonferroni post hoc test was performed. Spearman correlation analysis was performed between the propionate concentration and number of *E. coli* in pairwise comparisons. Differences corresponding to *P* values ≤ 0.05 were considered statistically significant.

## Supplementary Material

Supplemental Material
