# Supplementary material for: Propionate catabolism by CD-associated adherent-invasive E. coli counteracts its anti-inflammatory effect
Source: Gut Microbes. 2021 Mar 26;13(1):1839318. doi: 10.1080/19490976.2020.1839318 (PMC8007151; doi:10.1080/19490976.2020.1839318)
Supplement: Supplemental Material [file KGMI_A_1839318_SM5289.zip › Supplementary information/Supplementary captions.docx]

**Figure S1: Inverse correlation between fecal acetate concentration and the number of *E. coli* in the stool samples and ileal mucosa of Crohn’s disease patients.**

Inverse correlation between fecal acetate concentration and the number of *E. coli* bacteria associated with the ileal mucosa (A, C) and in the stool samples (B, D) from Crohn’s disease patients; AIEC+ (A, B) and AIEC- (C, D). Spearman correlation was performed between acetate concentration and number of *E. coli* in pairwise comparisons.

**Figure S2: Quantification of bacteria in stool samples (A) or associated with the colonic mucosa (B) showed no difference in colonization between the WT and mutant strains.**

CEABAC10 mice (n=8 for each group) were pretreated with fosfomycin (2 g/L) for 4 days and orally infected with the LF82 strain or the isogenic mutant LF82Δ*prpC*. Each symbol represents an individual mouse, and lines show medians.

**Figure S3: Propionate has no significant effect on AIEC LF82-related intestinal colonization and inflammation.**

CEABAC10 mice (n= 6 for each group) were pretreated with an antibiotic cocktail containing 500 mg/L metronidazole, 1 g/L streptomycin, 1 g/L neomycin and 1 g/L ampicillin. Mice received 1% DSS and, when needed, 0.6% calcium propionate in their drinking water in parallel with antibiotic treatment. On day 5, the antibiotic treatment was stopped; 0.25% DSS and 0.6% propionate or not were maintained in the drinking water. On day 6, mice were orally infected with AIEC LF82. (A-B) AIEC LF82 count in feces and intestinal mucosa of infected mice. (C) Administration of propionate did not modify the DAI score. (C-D) Administration of propionate did not impact the level of proinflammatory cytokines. Each symbol represents an individual mouse, and lines show medians. Statistical comparisons were carried out by normality testing using Kolmogorov-Smirnov tests, and a subsequent two-tailed Student’s test or Mann–Whitney U-test was performed.

**Figure S4: Quantification of bacteria in stool samples (A) or associated with the colonic mucosa (B) showed no difference in colonization between the WT and mutant strains.**

CEABAC10 mice (n= 9, two independent experiments pooled) were pretreated with an antibiotic cocktail containing 500 mg/L metronidazole, 1 g/L streptomycin, 1 g/L neomycin and 1 g/L ampicillin. Mice received 1% DSS and, when needed, 0.6% calcium propionate in their drinking water in parallel with antibiotic treatment. On day 5, the antibiotic treatment was stopped; 0.25% DSS and 0.6% propionate were maintained in the drinking water. On day 6, mice were orally infected with AIEC LF82 or the isogenic mutant LF82Δ*prpC.* Each symbol represents an individual mouse, and lines show medians.

**Figure S5: Analysis of epithelial differentiation and IEC proliferation in colonic mucosa of mice.**

CEABAC10 mice (n=8 for each group) were pretreated with an antibiotic cocktail containing 500 mg/L metronidazole, 1 g/L streptomycin, 1 g/L neomycin and 1 g/L ampicillin. Mice were orally challenged for 7 days with 10^9^ CFU of AIEC LF82 bacteria and with 5 mg/kg/day GPR43 agonist. Simultaneously, the drinking water of the mice was supplemented with 0.25% DSS. (A) Quantification of specific genes implicated either in the mechanism of epithelial differentiation (*Olfm4*, *Hes1*, *Atoh1* and *Muc2*), or IEC proliferation (*Pcna*, *CyclinD1* and *CyclinA*), in colonic mucosa of mice by RT-qPCR. (B) Colon crypt height of mice treated or not with the GPR43 agonist.

**Table S1:** **Primers used in this study.**

**Table S2: Disease Activity Index (DAI) scoring.**

**Table S3: Baseline characteristics of the 54 patients with CD enrolled in the study.**
