# Supplementary material for: Propionate catabolism by CD-associated adherent-invasive E. coli counteracts its anti-inflammatory effect
Source: Gut Microbes. 2021 Mar 26;13(1):1839318. doi: 10.1080/19490976.2020.1839318 (PMC8007151; doi:10.1080/19490976.2020.1839318)
Supplement: Supplemental Material [file KGMI_A_1839318_SM5289.zip › Supplementary information/Table_S2_DAI.pptx]

## Slide 1
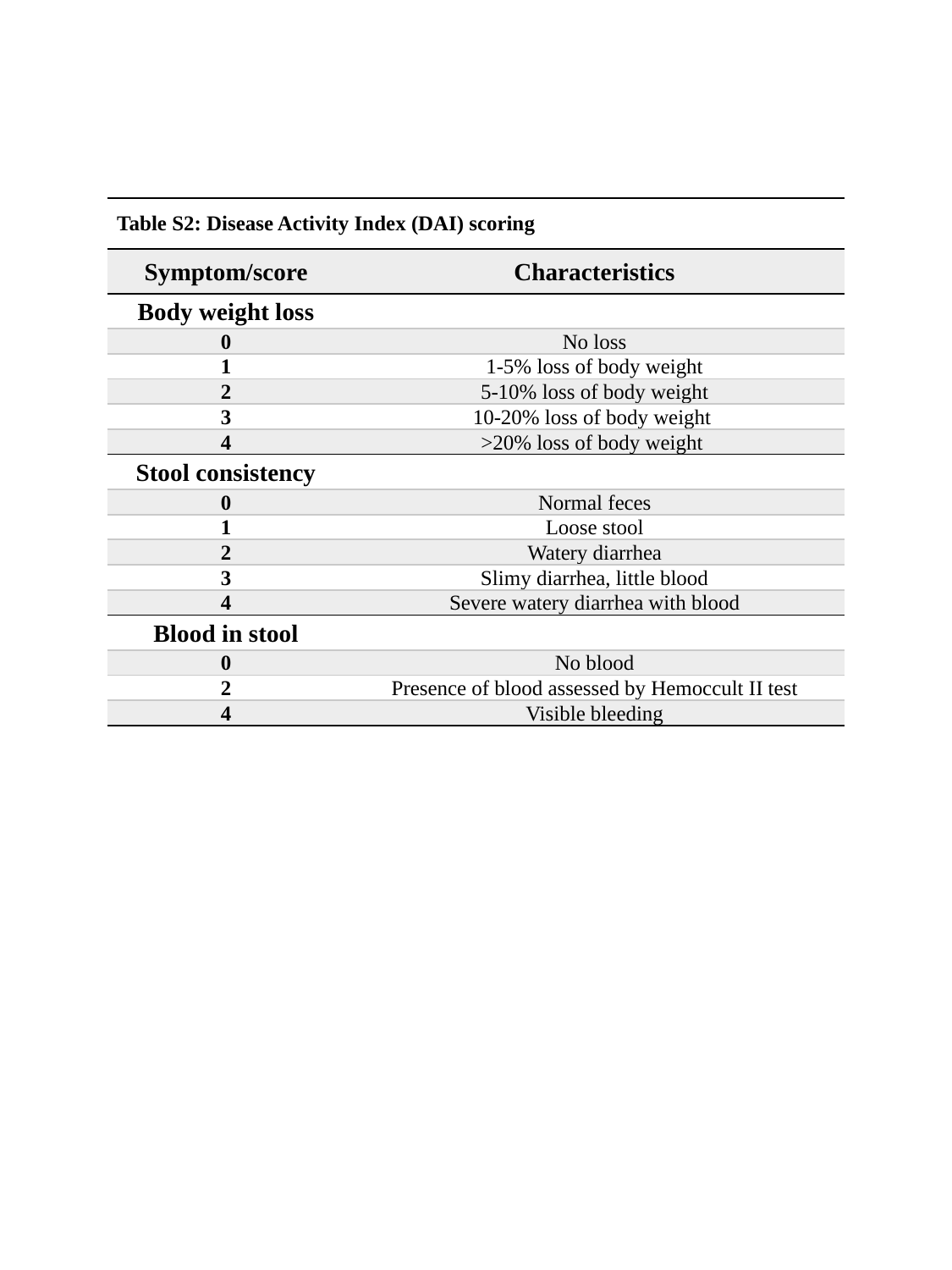

| Table S2: Disease Activity Index (DAI) scoring | |
| --- | --- |
| Symptom/score | Characteristics |
| Body weight loss | |
| 0 | No loss |
| 1 | 1-5% loss of body weight |
| 2 | 5-10% loss of body weight |
| 3 | 10-20% loss of body weight |
| 4 | >20% loss of body weight |
| Stool consistency | |
| 0 | Normal feces |
| 1 | Loose stool |
| 2 | Watery diarrhea |
| 3 | Slimy diarrhea, little blood |
| 4 | Severe watery diarrhea with blood |
| Blood in stool | |
| 0 | No blood |
| 2 | Presence of blood assessed by Hemoccult II test |
| 4 | Visible bleeding |
