# Supplementary material for: Propionate catabolism by CD-associated adherent-invasive E. coli counteracts its anti-inflammatory effect
Source: Gut Microbes. 2021 Mar 26;13(1):1839318. doi: 10.1080/19490976.2020.1839318 (PMC8007151; doi:10.1080/19490976.2020.1839318)
Supplement: Supplemental Material [file KGMI_A_1839318_SM5289.zip › Supplementary information/Table_S3_Patients_characteristics.pptx]

## Slide 1
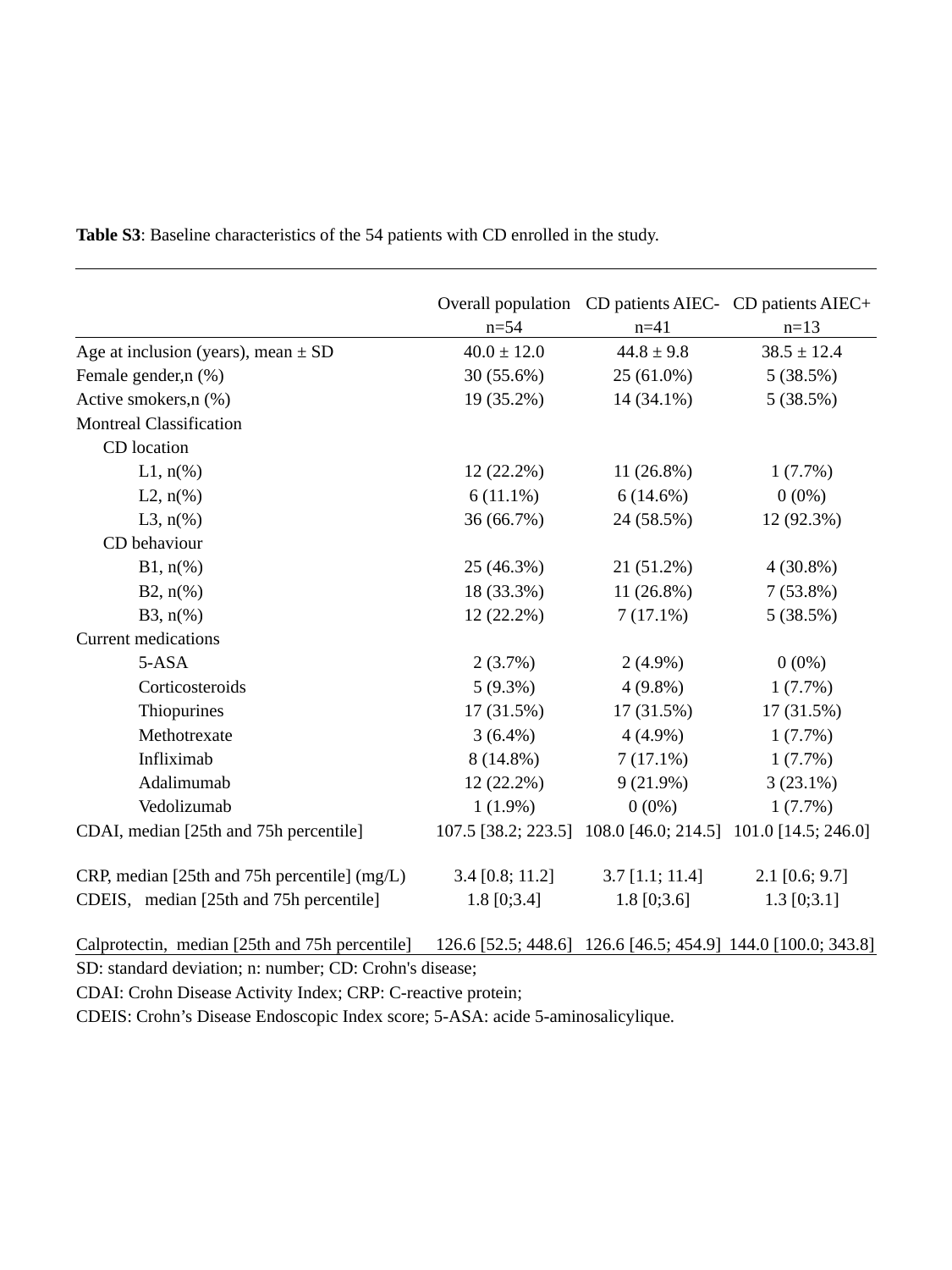

| Table S3: Baseline characteristics of the 54 patients with CD enrolled in the study. | | | | | |
| --- | --- | --- | --- | --- | --- |
| | | | | | |
| | | | Overall population | CD patients AIEC- | CD patients AIEC+ |
| | | | n=54 | n=41 | n=13 |
| Age at inclusion (years), mean ± SD | | | 40.0 ± 12.0 | 44.8 ± 9.8 | 38.5 ± 12.4 |
| Female gender,n (%) | | | 30 (55.6%) | 25 (61.0%) | 5 (38.5%) |
| Active smokers,n (%) | | | 19 (35.2%) | 14 (34.1%) | 5 (38.5%) |
| Montreal Classification | | | | | |
| CD location | | | | | |
| L1, n(%) | | | 12 (22.2%) | 11 (26.8%) | 1 (7.7%) |
| L2, n(%) | | | 6 (11.1%) | 6 (14.6%) | 0 (0%) |
| L3, n(%) | | | 36 (66.7%) | 24 (58.5%) | 12 (92.3%) |
| CD behaviour | | | | | |
| B1, n(%) | | | 25 (46.3%) | 21 (51.2%) | 4 (30.8%) |
| B2, n(%) | | | 18 (33.3%) | 11 (26.8%) | 7 (53.8%) |
| B3, n(%) | | | 12 (22.2%) | 7 (17.1%) | 5 (38.5%) |
| Current medications | | | | | |
| 5-ASA | | | 2 (3.7%) | 2 (4.9%) | 0 (0%) |
| Corticosteroids | | | 5 (9.3%) | 4 (9.8%) | 1 (7.7%) |
| Thiopurines | | | 17 (31.5%) | 17 (31.5%) | 17 (31.5%) |
| Methotrexate | | | 3 (6.4%) | 4 (4.9%) | 1 (7.7%) |
| Infliximab | | | 8 (14.8%) | 7 (17.1%) | 1 (7.7%) |
| Adalimumab | | | 12 (22.2%) | 9 (21.9%) | 3 (23.1%) |
| Vedolizumab | | | 1 (1.9%) | 0 (0%) | 1 (7.7%) |
| CDAI, median [25th and 75h percentile] | | | 107.5 [38.2; 223.5] | 108.0 [46.0; 214.5] | 101.0 [14.5; 246.0] |
| CRP, median [25th and 75h percentile] (mg/L) | | | 3.4 [0.8; 11.2] | 3.7 [1.1; 11.4] | 2.1 [0.6; 9.7] |
| CDEIS, median [25th and 75h percentile] | | | 1.8 [0;3.4] | 1.8 [0;3.6] | 1.3 [0;3.1] |
| Calprotectin, median [25th and 75h percentile] | | | 126.6 [52.5; 448.6] | 126.6 [46.5; 454.9] | 144.0 [100.0; 343.8] |
| SD: standard deviation; n: number; CD: Crohn's disease; | | | | | |
| CDAI: Crohn Disease Activity Index; CRP: C-reactive protein; | | | | | |
| CDEIS: Crohn’s Disease Endoscopic Index score; 5-ASA: acide 5-aminosalicylique. | | | | | |
