# Supplementary figures and images for: Propionate catabolism by CD-associated adherent-invasive E. coli counteracts its anti-inflammatory effect
Source: Gut Microbes. 2021 Mar 26;13(1):1839318. doi: 10.1080/19490976.2020.1839318 (PMC8007151; doi:10.1080/19490976.2020.1839318)

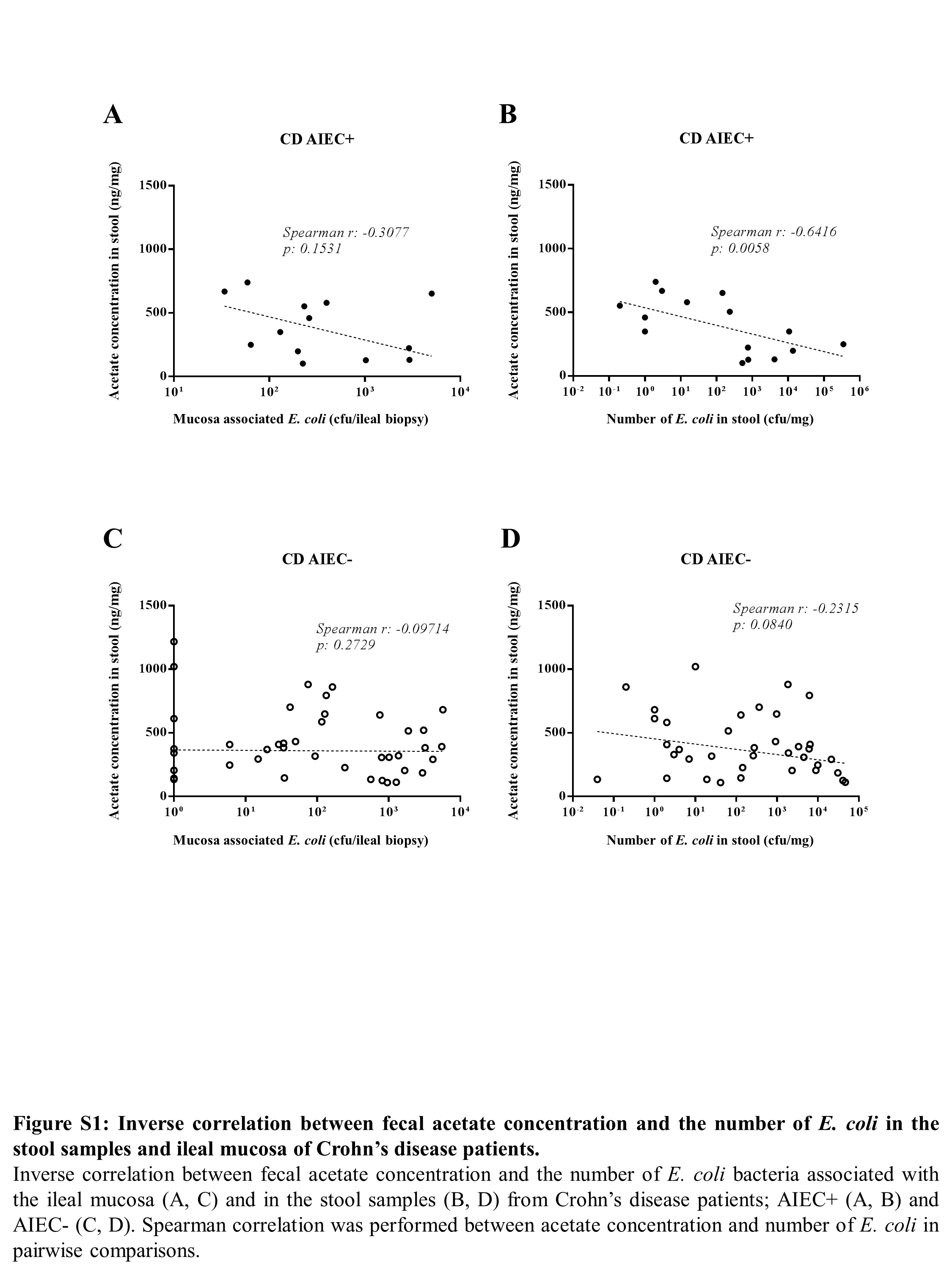

Supplement: Supplemental Material [file KGMI_A_1839318_SM5289.zip › Supplementary information/Figure_S1.tiff]

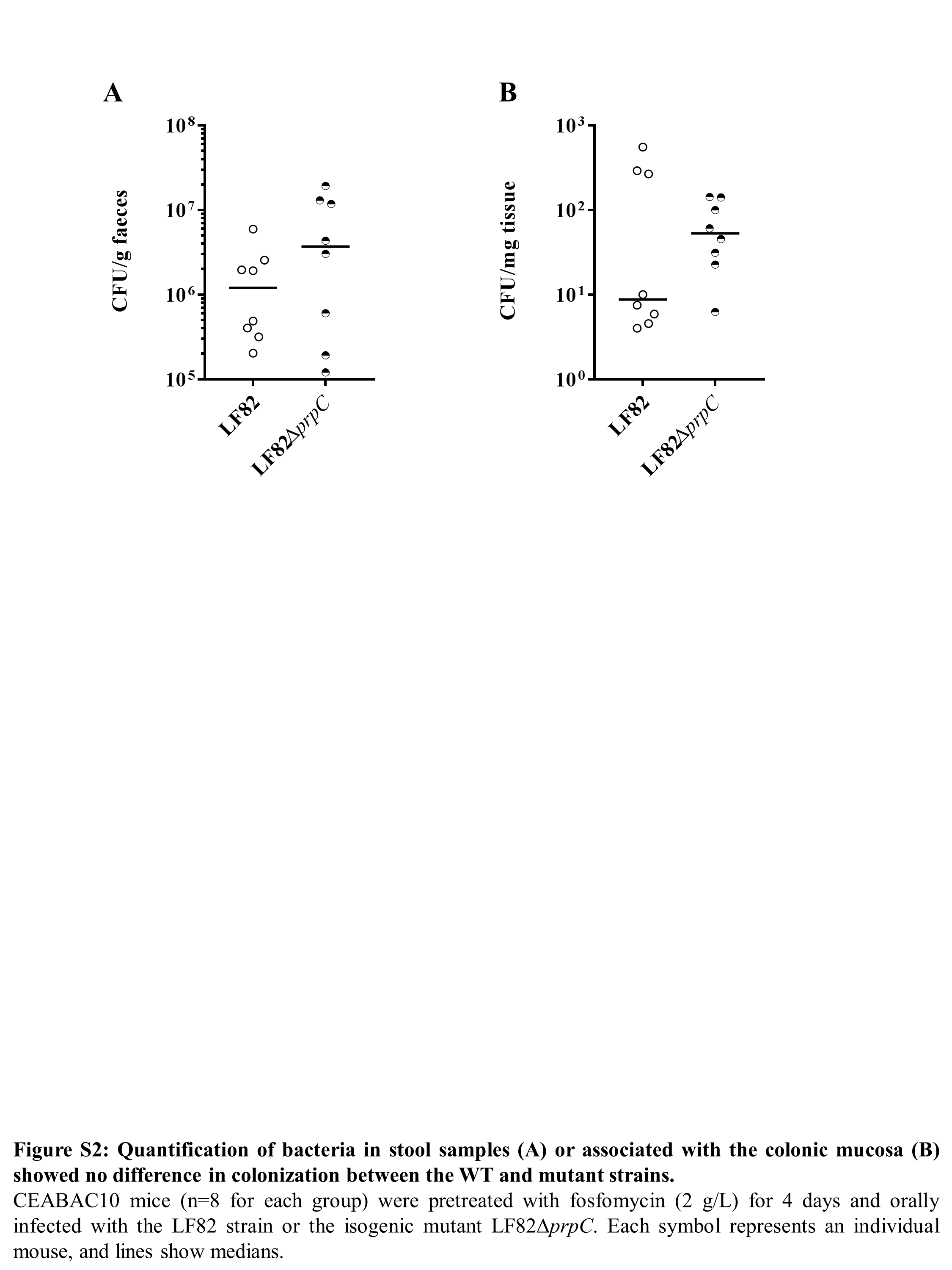

Supplement: Supplemental Material [file KGMI_A_1839318_SM5289.zip › Supplementary information/Figure_S2.tiff]

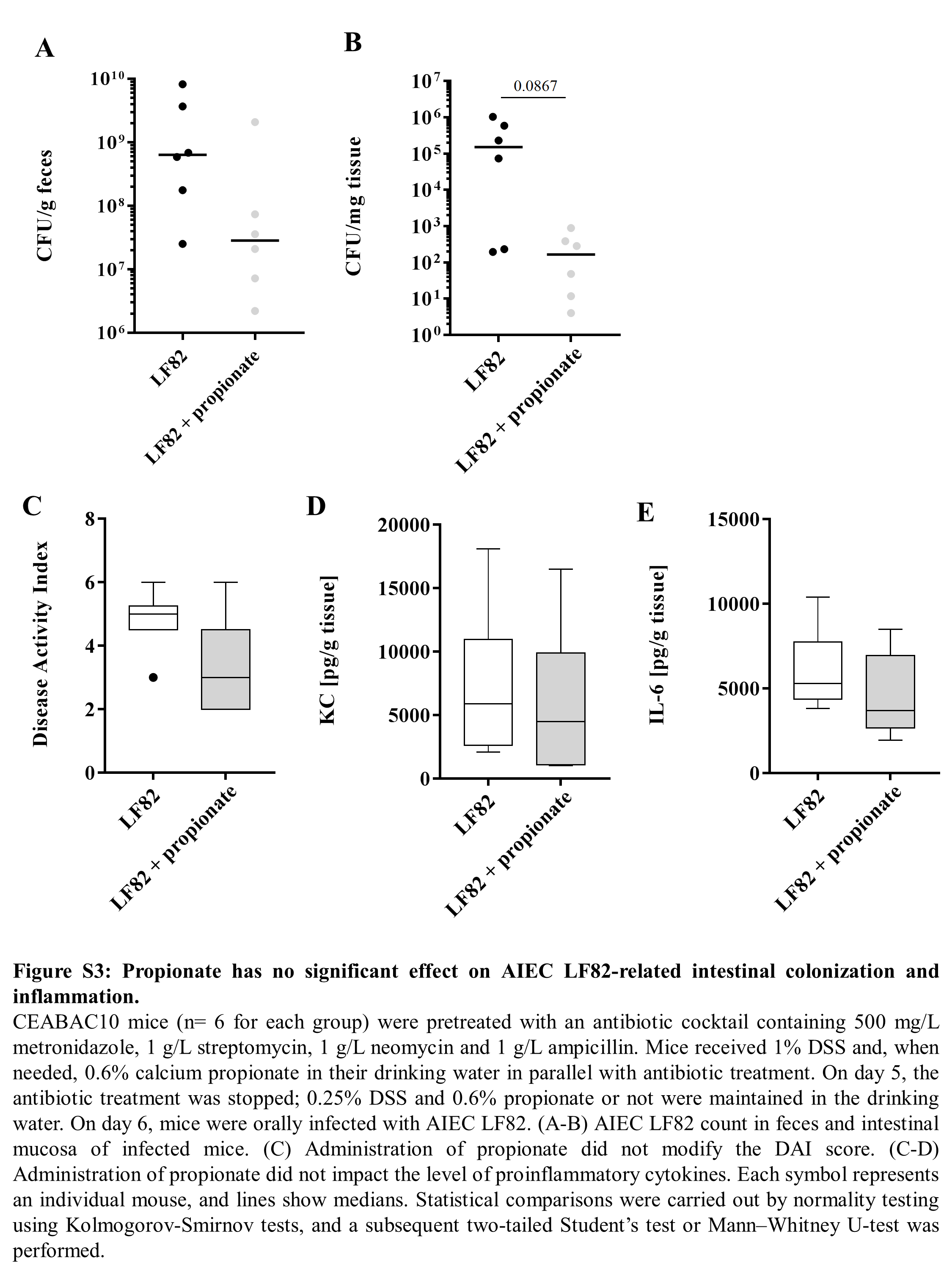

Supplement: Supplemental Material [file KGMI_A_1839318_SM5289.zip › Supplementary information/Figure_S3.tiff]

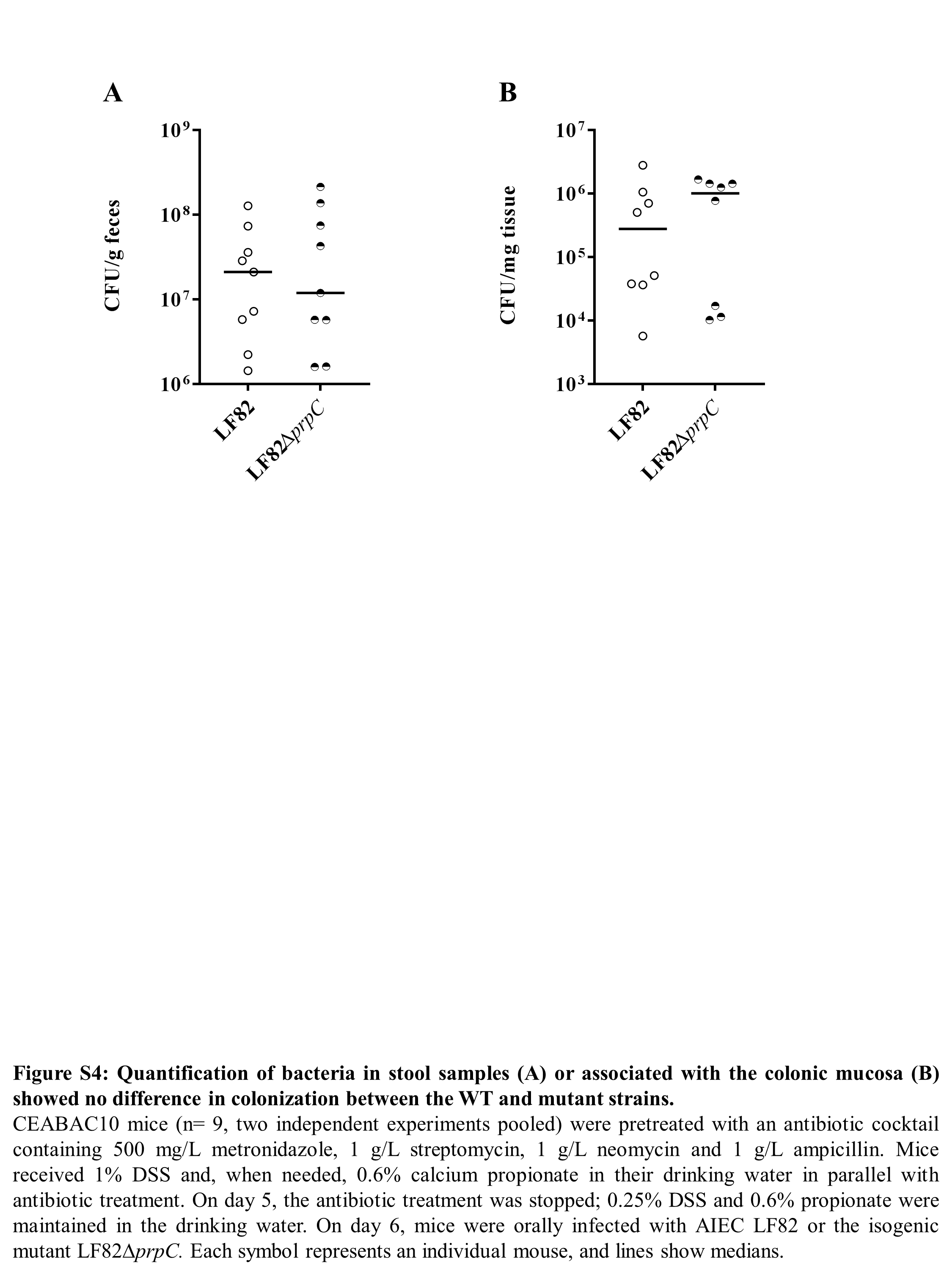

Supplement: Supplemental Material [file KGMI_A_1839318_SM5289.zip › Supplementary information/Figure_S4.tiff]

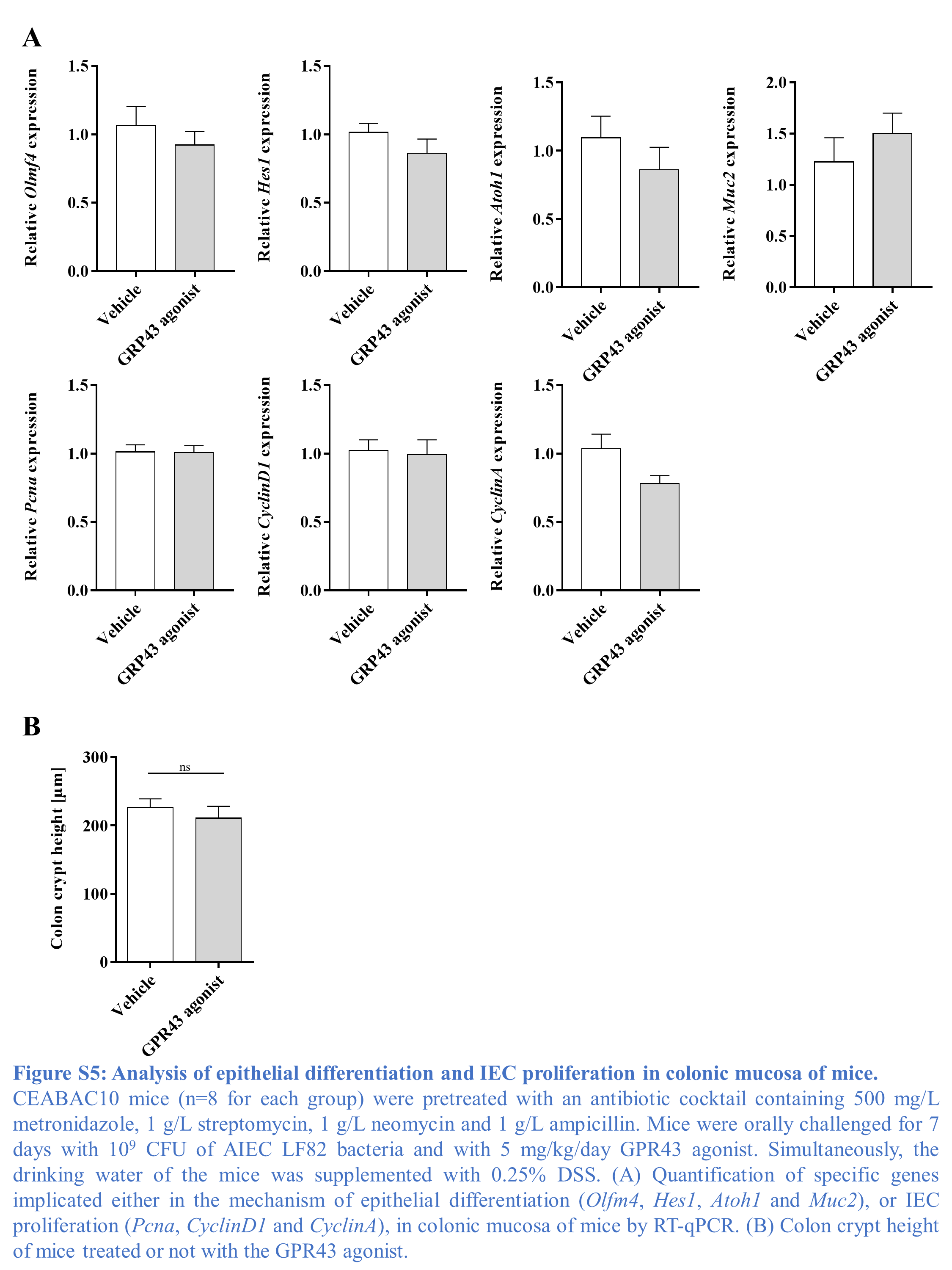

Supplement: Supplemental Material [file KGMI_A_1839318_SM5289.zip › Supplementary information/Figure_S5.tiff]

## Slide 1
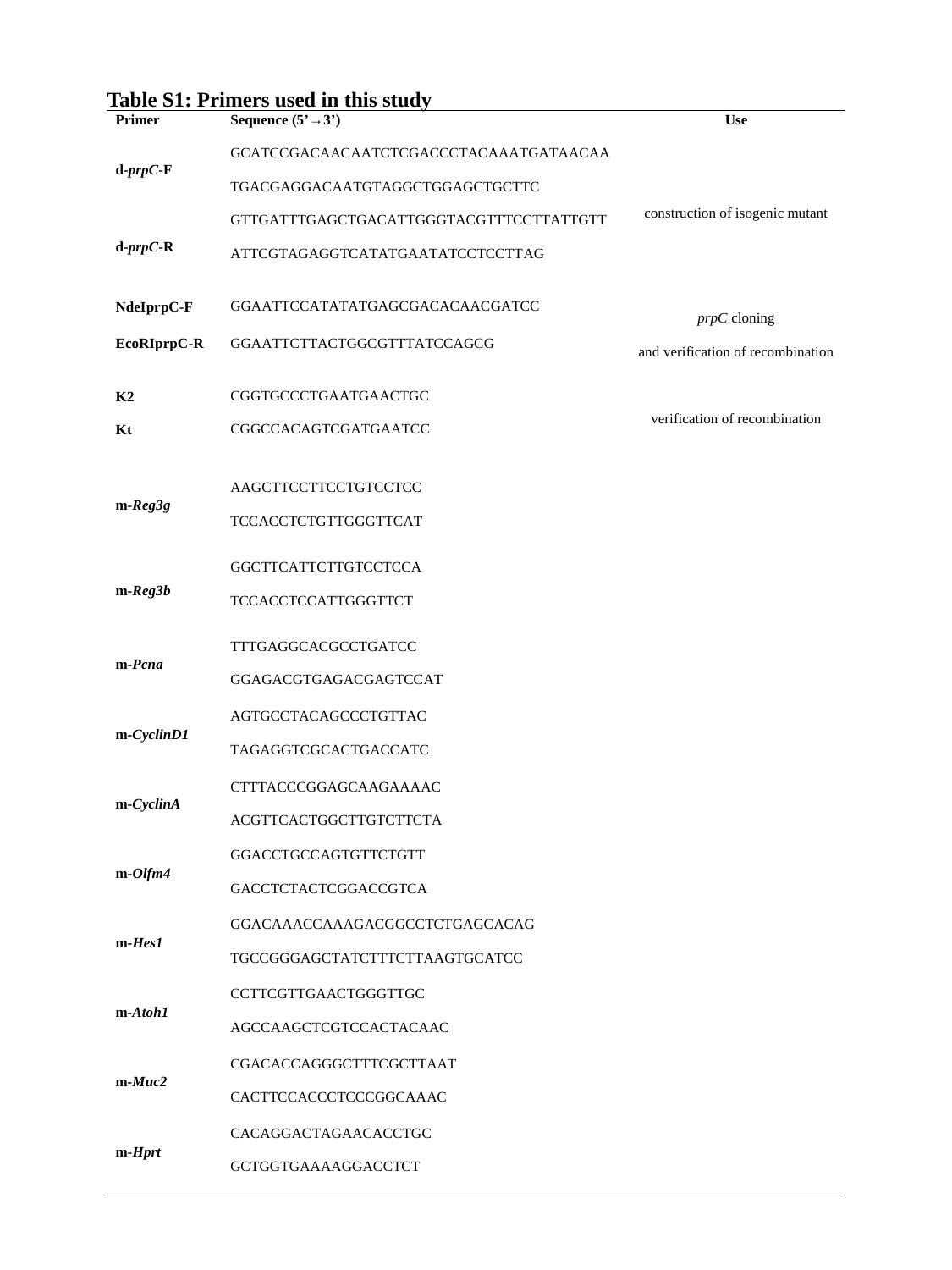

Table S1: Primers used in this study

Supplement: Supplemental Material [file KGMI_A_1839318_SM5289.zip › Supplementary information/Table_S1_Primers.pptx]
